# Emulating computer models with high-dimensional count output

**DOI:** 10.1098/rsta.2024.0216

**Published:** 2025-03-13

**Authors:** James M. Salter, Trevelyan J. McKinley, Xiaoyu Xiong, Daniel B. Williamson

**Affiliations:** ^1^ Department of Mathematics and Statistics, University of Exeter, Exeter, UK; ^2^ University of Exeter Medical School, University of Exeter, Exeter, UK; ^3^ Department of Economics, Land Environment Economics and Policy Institute, University of Exeter, Exeter, UK

**Keywords:** Gaussian processes, basis emulation, uncertainty quantification, Poisson lognormal

## Abstract

Computer models are used to study the real world, and often contain a large number of uncertain input parameters, produce a large number of outputs, may be expensive to run and need calibrating to real-world observations to be useful for decision-making. Emulators are often used as cheap surrogates for the expensive simulator, trained on a small number of simulations to provide predictions with uncertainty at unseen inputs. In epidemiological applications, for example compartmental or agent-based models for modelling the spread of infectious diseases, the output is usually spatially and temporally indexed, stochastic and consists of counts rather than continuous variables. Here, we consider emulating high-dimensional count output from a complex computer model using a Poisson lognormal PCA (PLNPCA) emulator. We apply the PLNPCA emulator to output fields from a COVID-19 model for England and Wales and compare this to fitting emulators to aggregations of the full output. We show that performance is generally comparable, while the PLNPCA emulator inherits desirable properties, including allowing the full output to be predicted while capturing correlations between outputs, providing high-dimensional samples of counts that are representative of the true model output.

This article is part of the theme issue ‘Uncertainty quantification for healthcare and biological systems (Part 1)’.

## 1. Introduction

Computer models can be used to simulate the real world and assess different future scenarios to aid decision-making, for example assessing non-pharmaceutical interventions in the UK at the start of the COVID-19 pandemic [1]. Such models may have large computational costs and many unknown inputs, and as such, it may not be feasible to properly explore the uncertainty in different scenarios. For decision-making, it is important to understand uncertainties in the output owing to unknown inputs, and calibrate models to real-world data so that future projections can be trusted and properly account for parameter uncertainty [2].

To enable more efficient exploration of the expensive model, cheap statistical emulators can be trained on a set of simulations and used as a fast approximation for the true model. Given a fast and accurate emulator, it is straightforward to explore substantially more of the input space and properly understand and quantify uncertainties in the model. Emulators have been widely used across many fields that use complex simulation models, including climate [3–5], biological systems [6,7], electrophysiology [8] and epidemiological and health-related applications, for example in modelling HIV [9], Ebola [10] and COVID-19 [11,12]. The emulated outputs may be single values or high-dimensional fields, with calibration to real-world observations (probabilistically [13,14] or by iteratively ruling out simulations that poorly match the data [3,4,7]) or sensitivity analysis (understanding which inputs and combinations of inputs are driving variability in the output [5,15]) often being the goal.

For agent-based, compartmental and other similar models that simulate the interactions of individuals or groups, the output is usually a high-dimensional set of counts that have dependence in space and/or time, with a stochastic nature owing to the randomness of interactions and the probability of e.g. transmitting an infection in the model [10]. In past COVID-19 applications, summaries of the output have often been used for emulation and calibration, partly owing to the availability of data for calibration, e.g. [11] emulation of deaths at national and regional levels on certain dates, and [16] calibrating to total number of cases at a national level for multiple countries.

In this article, the aim is to train an emulator that truly mimics the true expensive model, i.e. one that can produce correlated high-dimensional fields of counts. Although the focus here is on the emulation problem, having such an emulator has clear benefits for model calibration as correlations in the output are properly accounted for, and calibrating to summaries may hide competing localized biases that may be informative about the biases and inadequacies of the true model.

We emulate the high-dimensional count output of a COVID-19 simulator (MetaWards) by using a Poisson lognormal PCA (PLNPCA) basis decomposition [17]. In this model, a latent basis is constructed and latent vectors at any simulator input are emulated via a small number of coefficients, with the Poisson lognormal (PLN) model structure used to reconstruct fields of counts. By the construction of this basis, samples from the emulator are high-dimensional fields of counts that retain correlations from the training data, capturing the stochastic nature of the original model and allowing the emulator to produce output that is consistent with the structure and relationships in the original model for any setting of the inputs. We show that for the MetaWards example, this emulator predicts the output at unseen inputs well, and is competitive with directly emulating one-dimensional summaries of the output, with the most accurate emulator dependent on the particular training set and regional summary considered, suggesting that taking the approach of emulating all outputs at once while accounting for their correlated and count nature is effective.

Section 2 outlines Gaussian process (GP) emulation, a common tool for the emulation of computer models, in one dimension and via a basis decomposition for higher dimensional output. Section 3 describes the PLN decomposition method, and how this can be used to emulate high-dimensional count data. Section 4 describes the MetaWards model and data used in this study and §5 describes the training of emulators for MetaWards output, comparing the PLNPCA model to other approaches for different regional aggregations of the MetaWards output.

## 2. Emulation

In this section, we outline approaches to emulating different types of computer model outputs (one-dimensional, high-dimensional and stochastic). In each case, the aim is to train an emulator given simulations of the true, possibly computationally expensive, model (‘simulator’) of a real-world system at varied inputs, and use this emulator as an efficient representation of the simulator, with uncertainty as the true simulator has not been run at all possible inputs. The focus here is on emulation and predicting at unseen inputs, but given a suitable emulator, other typical tasks include sensitivity analysis and model calibration.

We assume that simulator f(⋅) takes a p-dimensional input vector x from parameter space $X\subset {\mathbb{R}}^{p}$, generating ℓ outputs that can be combined into a vector $f(\text{x})\in R^{\ell }$. For training emulators, we have n simulations at samples from X with output matrix $\text{F}=(f({\text{x}}_{1}),\ldots ,f({\text{x}}_{n}))$, where each column is a simulation at a different x, and each row is one of the ℓ simulator outputs.

### (a) Single outputs

For ℓ=1, whether the simulator f(⋅) returns only a single value, we are focusing on only a single output, or (parts of) a larger output field have been summarized to a single value, the output is often modelled as a GP [18,19]


$$f(\text{𝐱})|𝜷,\sigma^{2},𝝓,\sigma_{\epsilon }^{2}∼\text{GP}(m(\text{𝐱}),\kappa (\text{𝐱},{\text{𝐱}}^{\prime })),$$

defined by a mean function m(⋅), dependent on parameters β, and covariance function κ(⋅,⋅), dependent on variance $\sigma^{2}$, correlation lengths for each input dimension ϕ and nugget $\sigma_{\epsilon }^{2}$. The specifications of these are problem-dependent, and give the GP emulator great flexibility, with a natural provision of uncertainty. Training the GP on simulator data F gives


$$f(\text{𝐱})|\text{𝐅},𝜷,\sigma^{2},𝝓,\sigma_{\epsilon }^{2}∼\text{GP}(m^{*}(\text{𝐱}),\kappa^{*}(\text{𝐱},{\text{𝐱}}^{\prime })),$$

where $m^{*}$ and $\kappa^{*}$ are the conditional mean and covariance functions. By the properties of the GP, we can write down the expectation E[f(x)] and variance Var[f(x)] of the simulator output at any choice of x, with the variance increasing as the distance (in input space) from training points increases. If the nugget parameter $\sigma_{\epsilon }^{2}$ is equal to zero, the emulator interpolates the training data with zero variance, suitable for emulating deterministic simulators.

Where the output is stochastic, i.e. the output may be different if we run the simulator twice at the same input x, different assumptions may be required and [20] provides an overview and examples of emulating these types of models. In general, the GP is made heteroscedastic by adding parameter dependence to the nugget parameter, $\sigma_{\epsilon }^{2}(\text{x})$, instead of treating this as fixed (homoscedastic). If enough replicates at design points x are available, stochastic kriging can be used, where an additional GP is trained on the sample variance [21]. For non-normal output, instead of emulating the sample variance, GPs can be trained on chosen quantiles of the output at different values of x (quantile kriging, [10,22]). Alternatively, for cases with or without replicates, the stochasticity can be modelled by treating the variances at the training points as unknown parameters and estimating these (hetGP, [23,24]).

In all that follows, because replicated output will not always be available, we assume the use of hetGP to fit heteroscedastic GPs to stochastic simulator output, capturing the stochasticity via input-dependent nugget $\sigma_{\epsilon }^{2}(\text{𝐱})$.

### (b) Multivariate outputs

For ℓ>1, we may apply the above or similar approaches iteratively across all ℓ outputs [25,26], extend the covariance function to include inputs and outputs [27] or reduce the dimensionality of the output via its projection on to a basis representation, and fit one-dimensional emulators to this [13,14]. For example, by decomposing the simulator output F via its singular value decomposition (SVD), we have [13]


$$\text{𝐅}=(f({\text{𝐱}}_{1}),\ldots ,f({\text{𝐱}}_{n}))\in {\mathbb{R}}^{\ell \times n},\;(\text{𝐅}-𝝁{𝟙}_{n}^{T}{)}^{T}=\text{𝐔}\text{𝐃}{𝚪}^{T},$$

where $\mu \in R^{\ell }$ is the row-wise mean of F, $1_{n}\in R^{n}$ is a vector of 1 s, $\text{U}\text{D}\Gamma^{T}$ is the decomposition of the centred simulator output, and we can write simulator output f(x) as a linear combination of orthonormal basis vectors $\Gamma =(\gamma_{1},\ldots ,\gamma_{n})\in R^{\ell \times n}$,


(2.1) $$\begin{array}{lcr} & f({\text{𝐱}}_{j})=𝝁+\sum_{i=1}^{n}c_{i}({\text{𝐱}}_{j}){𝜸}_{i}+\epsilon ({\text{𝐱}}_{j})=𝝁+𝚪\text{𝐜}({\text{𝐱}}_{j})+\epsilon ({\text{𝐱}}_{j}), & \end{array}$$

where $c_{i}({\text{x}}_{j})$ is the coefficient given by projecting the jth simulation, $f({\text{x}}_{j})$, on to the ith basis vector, $\gamma_{i}$; and $\epsilon ({\text{x}}_{j})=0$ if $f({\text{x}}_{j})\in \text{F}$. In practice, as the vectors are ordered by the proportion of variability explained in F, this sum is truncated after q<n vectors such that a large percentage of F is explained. While this truncation means that $\epsilon ({\text{x}}_{j})\neq 0$ even for training data, q is chosen such that this error is relatively small compared with ensemble variability. For later basis vectors that explain low proportions of variability across F, there may be limited or no parameter signal, and so assuming these coefficients are independent of x and are zero-mean normal with a fixed variance, and accounting for them through the predictive variance, is usually suitable (described below).

To predict f(𝐱), therefore, by equation (2.1), we only need to predict the leading q coefficients from the projection on to a basis ${𝚪}_{q}$ (the first q columns of 𝚪), and can emulate each of these as in the single output case above, assuming independence of the coefficients on the orthogonal basis


$$c_{i}(\text{𝐱})|{𝜽}_{i}∼\text{GP}(m_{i}(\text{𝐱}),\kappa_{i}(\text{𝐱},{\text{𝐱}}^{\prime })),\;i=1,\ldots ,q,$$

where each of the q coefficient emulators can have different assumptions (mean, covariance, stationarity, etc.) with a different set of parameters ${𝜽}_{i}$ as required. As before, we can write the expectation and variance for each GP emulator and combine these across the q coefficients:


(2.2) $$\begin{array}{lcr} & \text{E}[\text{𝐜}(\text{𝐱})]=(\text{E}[c_{1}(\text{𝐱})],\ldots ,\text{E}[c_{q}(\text{𝐱})]{)}^{T},\;\text{Var}[\text{𝐜}(\text{𝐱})]=\text{diag}(\text{Var}[c_{1}(\text{𝐱})],\ldots ,\text{Var}[c_{q}(\text{𝐱})]). & \end{array}$$

From these, and via equation (2.1), we use the basis structure to write expectation and variance on the original ℓ-dimensional field for any 𝐱 as


(2.3) $$\text{E}[f(\text{x})]=\mu +\Gamma_{q}\text{E}[\text{c}(\text{x})],\;\text{Var}[f(\text{x})]=\Gamma_{q}\text{Var}[\text{c}(\text{x})]\Gamma_{q}^{T}+\Gamma_{-q}\Psi \Gamma_{-q}^{T},$$

where ${𝚪}_{-q}$ are the basis vectors after truncation and 𝚿 is a diagonal matrix where the diagonal elements correspond to the variance of the truncated coefficients [14]. To obtain posterior samples of f(𝐱), we can either sample from the ℓ-dimensional normal distribution given by equation (2.3), or sample from each coefficient emulator and map each sample back to the ℓ-dimensional field using equation (2.1).

With this method, we only need to train q≪ℓ individual GPs to represent the full field output, so the approach scales well for prediction and in downstream tasks such as calibration [28]. Samples from the emulator are also consistent with patterns in the training data, as each sampled full field f(𝐱) is a linear combination of the basis vectors, weighted by the emulated coefficients.

## 3. PLN basis

In this section, we consider emulating simulator output that consists of high-dimensional count data, as is often the case for agent-based and epidemiological models. Section 2 assumed normality and continuous output, and may provide reasonable emulators in some cases, e.g. if the counts are all far from zero, or if samples are not required to be integers, but in general may not be a suitable assumption to make. Basis structures have been used in emulating other types of high-dimensional non-continuous output, e.g. binary output [29].

Similarly to the multivariate approach above, we use a basis decomposition given by a PLNPCA model to decompose the simulator output and find a low-dimensional basis. We emulate the leading coefficients on this basis and use the PLN model to reconstruct high-dimensional fields while accounting for uncertainty from the coefficient emulators and the remaining basis vectors. The emulated coefficients are now on a latent basis, rather than relating to a linear combination of basis vectors representing the simulator output itself, but retain many of the benefits of a basis approach, e.g. fewer individual GPs to fit as q≪ℓ, leading to efficient sampling and samples that retain correlations from the training data.

### (a) PLN PCA

Given vectors $\left({\text{𝐲}}_{1},\ldots ,{\text{𝐲}}_{n}\right)$ with ${\text{𝐲}}_{i}\in {\mathbb{Z}}_{\geq 0}^{\ell }$, the PLNPCA model is written as [17]


$$\begin{array}{c} {\text{z}}_{i}∼N(\mu ,\Sigma ),\;\Sigma =\text{B}{\text{B}}^{T}\\ y_{ij}|z_{ij}∼Poi(\exp(z_{ij})),\;i=1,\ldots ,n,\;j=1,\ldots ,\ell \end{array}$$

for $𝝁\in {\mathbb{R}}^{\ell }$, $\text{𝐁}\in {\mathbb{R}}^{\ell \times r}$ and rank r≤ℓ, where the $y_{ij}$ are conditionally independent given $z_{ij}$. Alternatively, the ith latent vector can be written as


$${\text{𝐳}}_{i}=𝝁+\text{𝐁}{\text{𝐰}}_{i},$$

for latent coefficient vector ${\text{w}}_{i}\in R^{r}$, with $E[{\text{w}}_{i}]=0$ and $\text{Var}[{\text{w}}_{i}]=1$.

The identifiable parameters in this model are μ and $\Sigma =\text{B}{\text{B}}^{T}$, and given data $\left({\text{y}}_{1},\ldots ,{\text{y}}_{n}\right)$ these can be estimated via variational inference, performed here with R package PLNmodels [30]. Unlike in the normal SVD case, μ is no longer the mean of the training data, but a parameter estimated on the latent level (although the initial value may be set as the logarithm of the training data mean). In addition, B is not unique and in the estimation process is not constrained to be orthonormal, with only a constraint on its rank, r. However, given a fitted model, we have estimates of each latent vector ${\text{z}}_{i}$, and performing SVD on these latent values gives an orthonormal basis Γ with r vectors ordered by variance explained. Therefore, on the latent level, we have a decomposition analogous to the normal SVD case, with latent vectors ${\text{z}}_{i}$ decomposed into the sum of a common mean and a linear combination across orthogonal vectors.

Despite the conditional independence of $y_{ij}$, reconstructions of fields inherit patterns from the (latent) basis, so that sampling from the PLNPCA model can produce samples with similar correlations to the true output vectors. Owing to the PLN structure, we cannot predict negatives, and samples from the model (reconstructions of the original field from coefficients) are high-dimensional fields of counts, retaining correlations and properties from the original data. Samples can also be equal to zero.

### (b) PLNPCA and emulation

Rewriting the PLNPCA model with notation consistent with §2, the latent vector is now a basis representation of the ℓ-dimensional count field $f({\text{𝐱}}_{i})$, with dependence on simulator input ${\text{𝐱}}_{i}$. Given the simulator output $\text{𝐅}=\left(f({\text{𝐱}}_{1}),\ldots ,f({\text{𝐱}}_{n})\right)$, and an orthogonal latent basis $𝚪=({𝜸}_{1},\ldots ,{𝜸}_{r})\in {\mathbb{R}}^{\ell \times r}$, we can model the full output $f(\text{𝐱})\in {\mathbb{Z}}_{\geq 0}^{\ell }$ as


(3.1) (3)z(x)=μ+Γc(x),fj(x)|zj(x)∼Poi(exp⁡(zj(x))),j=1,…,ℓ,

where $\text{𝐜}(\text{𝐱})=(c_{1}(\text{𝐱}),\ldots ,c_{r}(\text{𝐱}){)}^{T}$ is the coefficient representation of f(𝐱) on the latent basis 𝚪. As with the normal SVD case, the basis can be truncated after the leading q<r vectors (i.e. those with dependence on 𝐱), with emulators trained for these q latent coefficients


$$c_{i}(\text{𝐱})|{𝜽}_{i}∼\mathrm{GP}(m_{i}(\text{𝐱}),\kappa_{i}(\text{𝐱},{\text{𝐱}}^{\prime })),\;i=1,\ldots q.$$

We assume heteroscedastic GP emulation for these one-dimensional outputs, as the latent coefficients have no restrictions on their values (continuous variables, centred at zero), and so the set of model parameters ${𝜽}_{i}$ includes an input-dependent nugget, giving the emulator more flexibility to capture stochasticity in the latent coefficients. The expectation and variance across the latent coefficients can be arranged analogously to the normal SVD case in equation (2.2), giving predictions of the ℓ-dimensional latent vectors at any 𝐱:


(3.2) $$\text{E}[z(\text{x})]=\mu +\Gamma_{q}\text{E}[\text{c}(\text{x})],\;\text{Var}[z(\text{x})]=\Gamma_{q}\text{Var}[\text{c}(\text{x})]\Gamma_{q}^{T}+\Gamma_{-q}\Psi \Gamma_{-q}^{T},$$

where as before, $𝚪=\left({𝚪}_{q},{𝚪}_{-q}\right)$ splits the basis into emulated and unemulated vectors, and 𝚿 is a diagonal matrix where the ith diagonal element is the variance of the latent coefficients on the (q+i)th basis vector. Overall, this looks similar to the normal SVD model, with the main difference being that emulation is now on a latent level, with this latent emulator output mapped back to the original scale via the Poisson distribution. Following [31], the expectation and variance of the simulator output at 𝐱 can be written by applying the PLN model (equation (3.1)) to the latent reconstruction as


$$\begin{array}{rl} \text{E}[f_{j}(\text{𝐱})] & =\exp(\text{E}[z_{j}(\text{𝐱})]+0.5\text{Var}[z_{j}(\text{𝐱})]),\\ \text{Var}[f_{j}(\text{𝐱})] & =\text{E}[f_{j}(\text{𝐱})]+\text{E}[f_{j}(\text{𝐱}){]}^{2}(\exp(\text{Var}[z_{j}(\text{𝐱})])-1),\\ \text{Cov}[f_{j}(\text{𝐱}),f_{k}(\text{𝐱})] & =\text{E}[f_{j}(\text{𝐱})]\text{E}[f_{k}(\text{𝐱})](\exp(\text{Cov}[z_{j}(\text{𝐱}),z_{k}(\text{𝐱})])-1),\;j\neq k, \end{array}$$

with the correlation structure in the simulator output imposed through the latent basis and transformation of the latent variables to the response. By sampling from the GPs on the coefficients, we can obtain samples of the latent vector z(𝐱) for any input 𝐱, and via the PLN model, can sample values of the true high-dimensional count field f(𝐱).

In the PLN model, the expectation of f(𝐱) is not simply the mean of the Poisson distribution but is also dependent on the variance at the latent level. Similarly, the variance is not equal to the mean. As the rank r is a choice when constructing the basis 𝚪, while it may not have a large effect on the leading parameter-dependent vectors, it affects the number of deleted basis vectors, and hence the inclusion of these in equation (3.2) would appear to inflate the latent variance, Var[z(𝐱)], and hence affect E[f(𝐱)]. However, changing r affects the estimation of 𝝁,𝚺, so that this ‘extra’ variance has been accounted for in the construction of the PLNPCA model, and these deleted vectors are required to reconstruct the correct expectations on the original field.

Unlike normal SVD, there is not a projection equation for determining the basis coefficients of any generic output field, and instead, we have to approximate out-of-sample latent representations. However, given emulators for the leading q coefficients, we can write down the expectation and variance of the ℓ-dimensional latent vector at any 𝐱, and hence either write down the properties of f(𝐱) or sample fields. For validating emulators out-of-sample, we do not compare on the coefficient level, but instead assess emulator performance on the original scale by sampling or via the expectation and variance properties of the model, and compare these to the true simulator counts.

## 4. The MetaWards simulator

MetaWards [32,33] is a stochastic metapopulation model for simulating the spread of a disease in Great Britain. The model uses population at an electoral ward level split into different age groups, with the movement of individuals in the model controlled by a contact matrix that describes which wards they travel to during the day. The model structure here has eight compartments: S (susceptible), E (exposed), A (asymptomatic), P (pre-symptomatic), I (infectious, symptomatic), H (hospitalized), R (recovered) and D (died).

MetaWards has previously been used to simulate the spread of COVID-19 in England and Wales [34], and we use it for this purpose here. In our study, p=15 input parameters are varied (described in electronic supplementary material, table S1), controlling aspects of the transition through the eight classes, for example the mean time that a person is in the E class ($T_{E}$) and the probability of transitioning from class H to class D ($\alpha_{HD}$); parameters that control the impact of age ($\eta ,\eta_{T_{H}}$) and the reproduction number ($R_{0}$). All of these could be estimated from data; however, uncertainty exists, and their prior distributions are assumed here to be reasonably wide to properly capture what may be the ‘true’ values and allow the exploration of possible scenarios. For example, the reproduction number $R_{0}$ is varied between 2 and 4.5.

In the ensemble used in §5, the simulation has been run up to immediately prior to the first lockdown (23 March 2020), initialized 12 weeks earlier and seeded with a small number of initial cases, with a scaling parameter $n_{s}$ to reflect that the initial seeding is uncertain and will affect the results. There are a total of n=250 unique samples from the 15-dimensional parameter space X. Of these, 200 have a single simulation, and 50 have been replicated 10 times.

The model tracks the number of individuals in each class by ward over time, and we focus on the total number of deaths at the end of the simulation, for different spatial aggregations. From the 8071-dimensional ward-level output, we aggregate to local authority district (LAD) level (339 outputs), regional level (9 outputs) and national level (1 output), with a goal of accurately emulating the count output at different spatial resolutions.

## 5. Results

In this section, we emulate the total deaths at the end of the MetaWards simulation described above, fitting emulators for high-dimensional count output at the LAD and ward levels. We show examples of the PLNPCA model applied to training data without and with replicates, as depending on model expense and other factors, replicates may not always be available. We also consider different sizes and compositions of the training set.

As a benchmark, we emulate regional and national summaries of the output with individual emulators. For comparing methods, we aggregate the basis emulators to regional and national totals by sampling from the coefficient emulators at an input 𝐱 and from these reconstructing full fields. We can then compare the mean and 95% intervals of these predictions to those given by an emulator trained on the national and regional totals explicitly. The goal of this comparison is to assess whether the additional complexity of the basis emulators, attempting to account for all of the spatial information simultaneously, is causing much predictive ability to be lost if we only consider totals instead. Emulating all 339 (LADs) or 8071 (wards) outputs at once is unlikely to outperform directly emulating totals, but how much accuracy is lost as a trade-off for gaining extra granularity in the emulator is unclear. We show that little accuracy is being lost by the PLNPCA approach, while gaining benefits, including being able to predict and sample high-dimensional fields of spatial counts at new 𝐱 having trained only q≪ℓ GPs, and with these predictions reproducing correlated structure from and hence better representing the original simulator.

In all cases, the validation set is neither used in the construction of the basis nor in the construction of the emulators: these runs are completely out-of-sample. The PLNPCA basis is calculated via PLNmodels. All GPs are fitted via hetGP [24] as the output, whether a summary of f(𝐱) or a basis coefficient, is stochastic.

### (a) Emulation without replicates

Here, we show in more detail a particular application of the PLNPCA emulator. We initially ignore replicates and only use a single simulation from each of the 250 inputs 𝐱. The training set consists of n=200 runs, and we predict for the remaining 50 inputs. The PLNPCA basis is trained on both LAD-level (PLNPCA-LAD) and ward-level (PLNPCA-ward) output. In both cases, we calculate the basis using the 200 training samples, fit GPs to the leading q=3 latent coefficients and from these predict at the 50 validation inputs. Given the mean and variance at 𝐱 for each coefficient, we sample latent fields z(𝐱) and hence counts f(𝐱) via the PLNPCA structure (equation (3.1)). The resulting samples of counts are either 339- or 8071-dimensional, and each can be aggregated in different ways to compare to the true output. Rather than validation via aggregation, we can also validate the individual GPs, e.g. by looking at leave-one-outs on the latent coefficients; however comparing the aggregations is more intuitive, and can be compared with other, non-latent, emulators. Electronic supplementary material, figs. S1 (LAD) and S2 (ward) shows the leading latent basis vectors, and these exhibit correlation.

**Figure 1.**
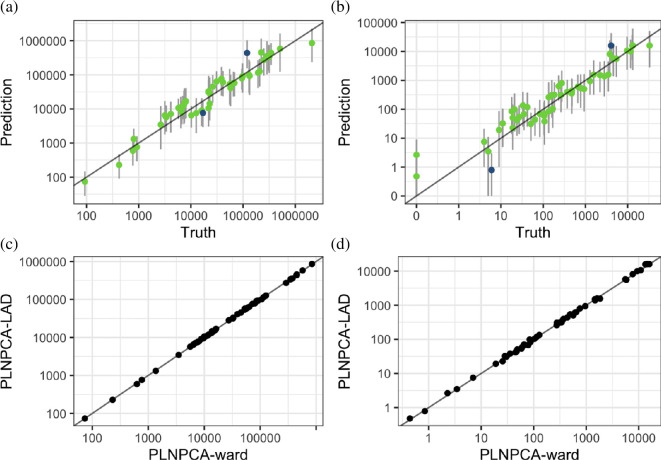
(*a*) and (*b*) Predictions for the 50 validation inputs from the PLNPCA-LAD hetGP emulator, aggregated to the overall total (*a*) and the northeast region (*b*), plotted against the true simulator output. Points are coloured green if the truth lies within the 95% error bars, blue otherwise. (*c*) and (*d*) Comparing mean predictions from the PLNPCA-LAD and PLNPCA-ward hetGP emulators for the overall total (*c*) and northeast region (*d*).

Figure 1*a*,*b* shows PLNPCA-LAD emulator predictions of the overall deaths (figure 1*a*) and deaths in the northeast (NE) region (figure 1*b*), aggregated from samples at the 50 validation points. The NE region is highlighted here as the region with output least correlated with the overall total. Both demonstrate that the emulator is predicting well out-of-sample, despite the fact we are not directly emulating either of these quantities. Figure 1*c*,*d* plots the mean predictions for the total (figure 1*c*) and NE region (figure 1*d*), for PLNPCA-LAD and PLNPCA-ward. Despite disaggregating the output from 339 LADs to 8071 wards, and therefore needing to estimate a much higher dimensional basis from the same number of training points, emulator performance is relatively similar, out-of-sample. Electronic supplementary material, figs. S3–S6, show plots for the other regions.

**Figure 2.**
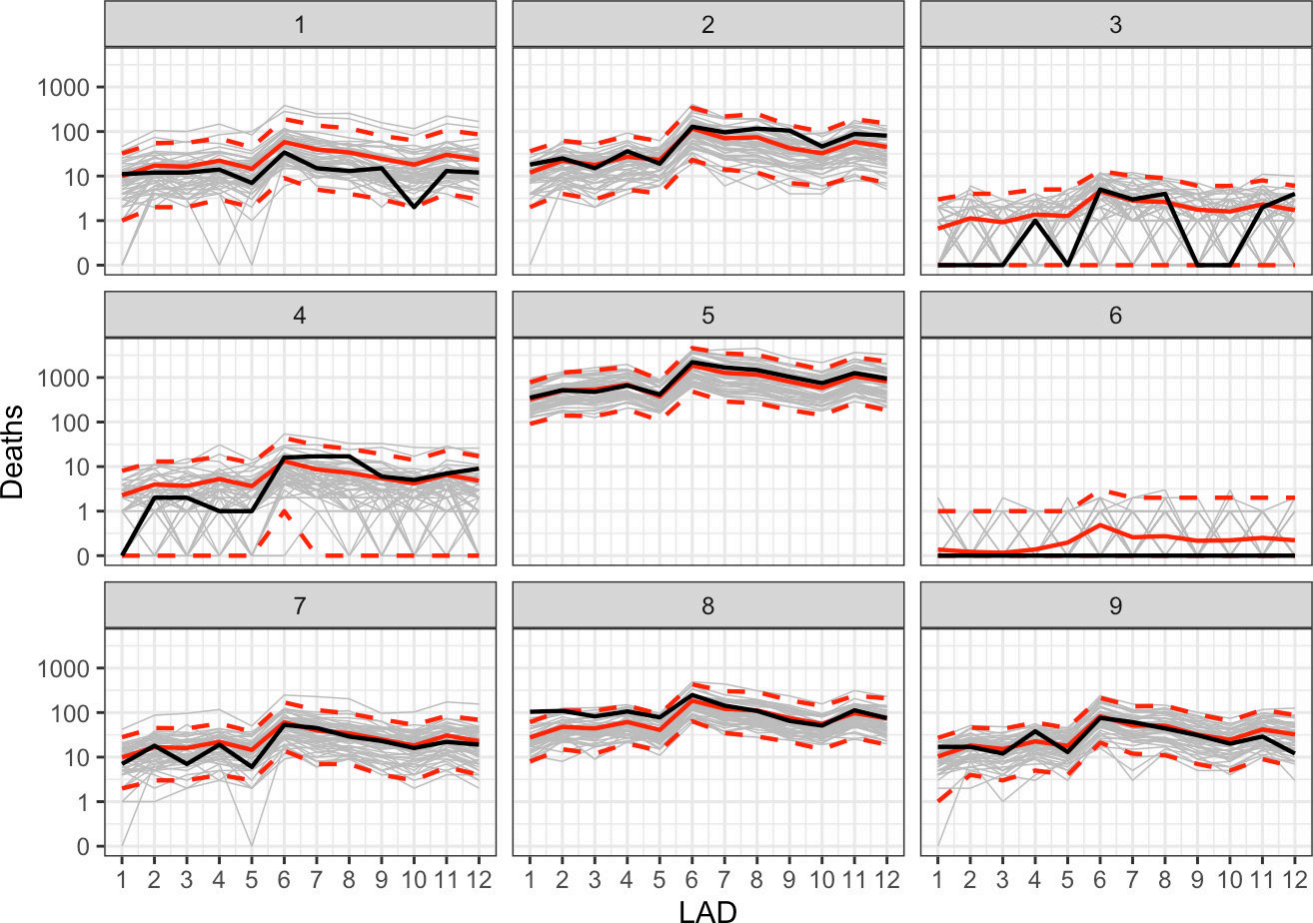
Samples from the PLNPCA-LAD hetGP emulator for nine validation inputs across the 12 LADs in the northeast region. For each, 50 individual samples are plotted in grey, with the mean (solid line) and 95% interval (dashed lines) across 1000 samples in red and true simulator output in black.

A benefit of these emulators is that we are not restricted to aggregations only, and can view predictions or samples at the LAD or ward level. Figure 2 shows samples from the PLNPCA-LAD emulator across the 12 LADs in the NE region, with each panel representing an individual validation input 𝐱, with true simulator output (black), emulator samples (grey) and their mean and 95% interval (red). The uncertainty on these plots is capturing both the uncertainty owing to extrapolation to unseen 𝐱∈X, and the uncertainty owing to the stochastic nature of the simulator. In this region, the simulator output spans multiple orders of magnitude, with some simulations having zero deaths (panel 6) and others having over 100 per LAD (panel 5). The emulator broadly captures the true magnitude for each validation point, with the majority of samples equal to 0 or 1 for input 6. Electronic supplementary material, figs. S7–S23, contain plots for other regions and the PLNPCA-ward emulator.

**Figure 3.**
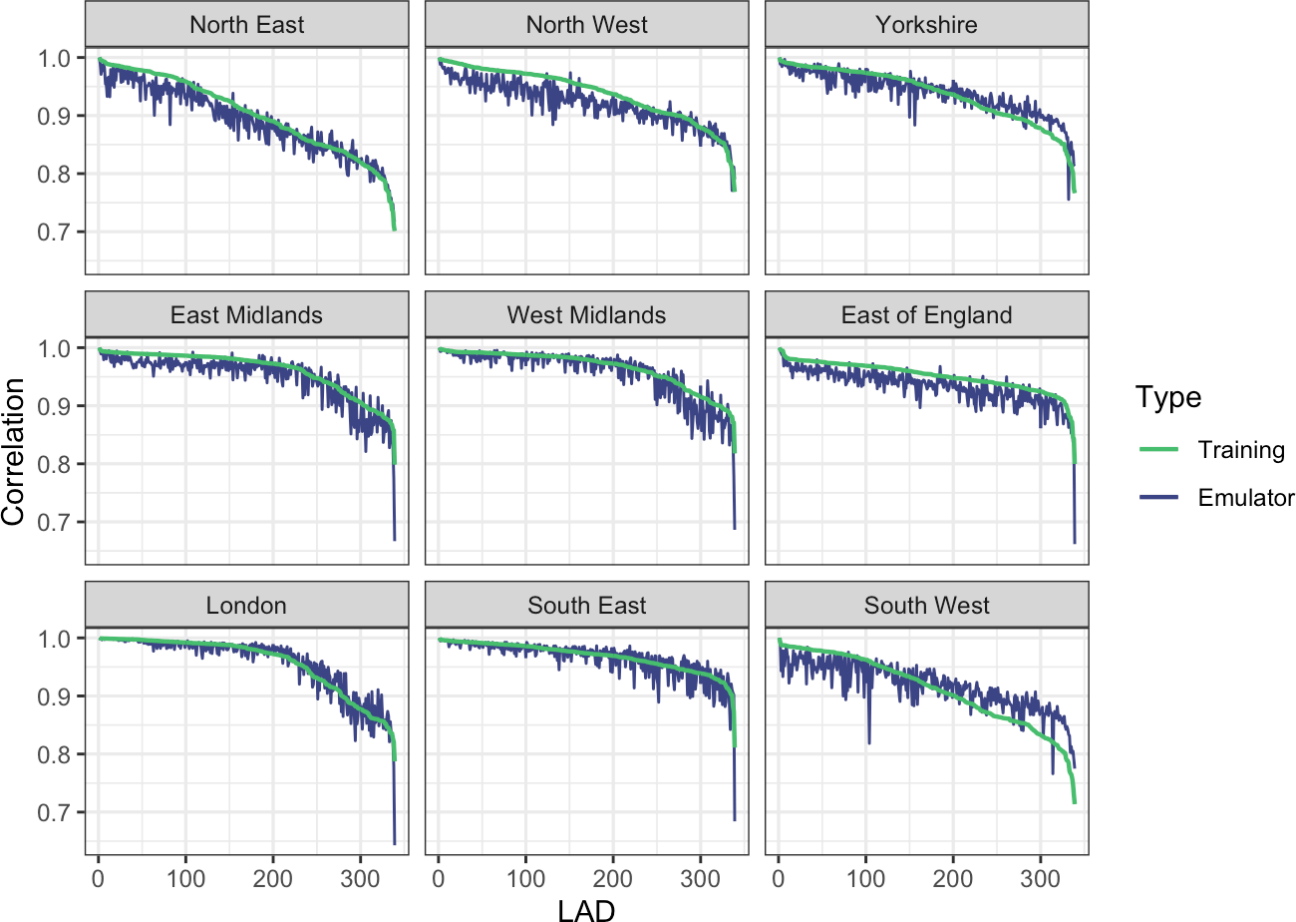
Comparing LAD-level correlations in the training data and from the PLNPCA-LAD emulator. For each region, a single LAD was randomly chosen, and the correlations between this LAD and all 338 others in the training data and emulator samples calculated. In each panel, the LADs are ordered from most to least correlated in the training data.

The predictions from the emulator overall seem to be accurate, but with the same set of samples (i.e. without needing to train a new emulator), we can produce comparisons for any other combination of LADs/wards or for individual locations. For example, we can assess whether samples capture the correlated structure in the true simulator output. Figure 3 plots the correlation between the number of deaths in (i) a LAD chosen randomly for each of the nine regions and (ii) the other 338 LADs, in both the training data and PLNPCA-LAD emulator samples. The emulator samples are maintaining the relationships seen in the true data well. Part of this may be due to the fact that there are relatively strong correlations across the output, but the emulator is generally retaining the relative relationships between different LADs, with the stronger and weaker correlations seen in the true data generally retained by the emulator. One of the motivating factors behind this emulator was that samples drawn from it should be able to maintain correlation information so that the true simulator and uncertainties can be more accurately represented by sampling from the emulator. This plot suggests that this works and is a feature that only emulating totals or each output independently does not have.

Calculation of the PLNPCA basis is more expensive than standard SVD (electronic supplementary material, table S3) and depends on dimension ℓ, number of simulations n and rank r; however given a fitted basis, to predict counts across ℓ locations requires only q fitted emulators. Compared with emulating all outputs separately, this saves training and validation time and compared with SVD, better represents the nature of the simulator.

### (b) Emulation with replicates

**Figure 4.**
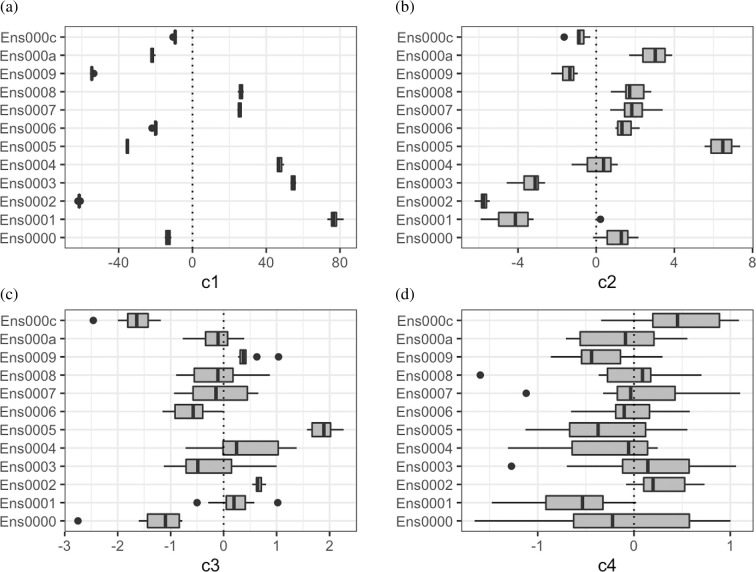
Latent coefficients $c_{1},c_{2},c_{3},c_{4}$ on the PLNPCA-LAD basis for 12 of the ensemble members with 10 replicates. The box plots show the variability in coefficient values across the replicates of these 12 input vectors.

We repeat the same modelling as in §5a, with replicates added for inputs where these were available. With replicates available, the variability on the latent basis may give information of which basis patterns contain signal from 𝐱, and which represent random variability in the simulator. For example, we can consider the mean and variance of coefficients across replicates at 𝐱 on each basis vector ${𝜸}_{i}$, and these may be informative about where we can truncate the basis. If the mean of a coefficient is always around zero across replicated simulations, it is likely that this vector represents a pattern owing to stochasticity rather than changes in 𝐱. Figure 4 shows the variability across the first four latent coefficients for 12 ensemble members that were replicated 10 times. On the first basis vector (figure 4*a*), there is a clear parameter signal, with the range of coefficient $c_{1}$ relatively narrow for a given 𝐱, compared with the overall range of $c_{1}$. For the second and third coefficients, the variance at a given 𝐱 is still lower than the overall variance and hence there is still probable dependence on 𝐱; however, by $c_{4}$, all of the 12 ensemble members plotted have distributions containing zero, and the variability for a given 𝐱 is not dissimilar from the overall variability on this coefficient. For this coefficient (and later ones, electronic supplementary material, fig. S24), we may no longer be identifying parameter signal, but random variability owing to the stochastic nature of the simulator, although we should attempt to emulate this coefficient as a further check.

Here, we truncate at q=3 as emulating $c_{4}$ led to little predictive ability. The remaining basis vectors are accounted for in the emulator variance (equation (3.2)), and hence samples from the emulator account for these as samples from a zero-mean normal. As figure 4 and attempts to emulate the fourth coefficient appear to suggest that this pattern is not representing parameter dependence but random noise, this is reasonable, although conversely, this may suggest that to identify parameter relationships on this vector, we need even more replicates. Either way, being able to account for this randomness in the emulator by sampling ensures that the emulator more accurately represents the true stochasticity of the simulator.

Electronic supplementary material, figs. S25–S38 in the supplementary material show validation plots and samples from the emulator trained using replicates, compared with the true output. These are similar to the without-replicate versions, suggesting that the emulators in the previous example were successfully identifying the stochastic part of the output, despite only seeing a single simulation at a given 𝐱.

### (c) Repeated experiments

To compare emulation approaches, we randomly split the 250 unique choices of 𝐱 into training and validation sets, for 50 training sets of sizes n=100,150,200. For each training set, we fit emulators with basis approaches and by directly emulating aggregations of the output, and predict across the validation set. For the PLNPCA approaches, we emulate the leading q=3 basis vectors as subsequent vectors are generally not 𝐱-dependent (§5*b*). For efficiency, we estimate a basis with r=10, as testing suggested that a higher r did not have a significant effect on the leading basis vectors or on the predictive variance, i.e. having additional deleted basis vectors does not have a large effect, as this is accounted for in the basis estimation process. For directly emulating the national/regional counts, we model the logarithm as this is more comparable with the PLNPCA version, and owing to the exponential dependence on the $R_{0}$ parameter, it is generally more accurate. Similarly, when using a normal SVD basis, we first take the logarithm of the output, as not doing so resulted in very negative values.

Table 1 compares directly emulating the overall and regional totals (‘aggregated’) with inferring these totals from a basis decomposition and emulated coefficients, either over LADs (‘SVD-LAD’, ‘PLNPCA-LAD’) or wards (‘PLNPCA-ward’). To compare, we sample from the basis emulators and aggregate across the corresponding LADs or wards. The table lists the percentage change in median RMSE for the validation set compared with a baseline of emulating aggregated output directly with hetGP; the percentage of experiments where an emulator type has lower RMSE than the baseline emulator and the median percentage of true values within 95% prediction intervals for the validation set. Table 1 also lists the results for the total and nine regions across the 50 experiments with n=200 (electronic supplementary material, tables S5 and S6 give results for n=150,100, respectively).

**Table 1.** Summaries of the results across 50 experiments for n=200, for the total output and each of the nine regions, for different emulators. ‘RMSE’ gives the percentage change in the median results compared with the hetGP emulator for the aggregated output; ‘RMSE < baseline’ gives the percentage of experiments where each emulator outperforms the aggregated version; ‘In95’ gives the median percentage of true values contained within 95% prediction intervals.

Metric	Emulator	All	NE	NW	YO	EM	WM	EE	LO	SE	SW
RMSE (% change)	aggregated	—	—	—	—	—	—	—	—	—	—
	SVD-LAD	22	13	20	17	19	16	25	16	21	23
	PLNPCA-LAD	7	−5	5	−3	4	7	7	4	7	6
	PLNPCA-ward	7	−5	7	−3	3	7	8	4	8	11
RMSE < baseline (%)	aggregated	—	—	—	—	—	—	—	—	—	—
	SVD-LAD	0	6	2	2	0	2	0	2	0	0
	PLNPCA-LAD	6	62	16	76	32	18	8	18	10	18
	PLNPCA-ward	6	56	12	72	28	16	4	16	12	10
In95 (%)	aggregated	98	94	98	94	98	98	98	97	98	98
	SVD-LAD	96	94	96	96	96	94	96	94	96	96
	PLNPCA-LAD	96	96	96	96	94	95	95	96	96	96
	PLNPCA-ward	96	94	96	96	95	94	96	96	96	94

To produce the results for the table, the aggregated-output version requires each regional total and the overall to be emulated independently, for a total of 10 emulators per training set. For the basis versions, we only need to fit q=3 emulators on the coefficients, and from this can sample and aggregate to any total of interest, or indeed any individual location, without needing to refit the emulator. The main expense comes from calculating the basis, but given this, emulation and prediction are efficient and encode correlations.

Considering the two PLNPCA versions and hetGP emulation of the totals, there is a 7% increase in median RMSE for the overall total when using a basis, with all three of these emulators having approximately 95% of predictions in 95% intervals on average. Emulating one output instead of 339 or 8071 is unsurprisingly more accurate in general if we target a summary, but is not true for every training/validation set split: for 6% of training sets, the PLNPCA approaches result in more accurate emulation of the total than the baseline emulator. Furthermore, the same PLNPCA emulators lead on average to greater accuracy for NE (5% better) and Yorkshire and the Humber (YO, 3% better), and have lower RMSE in over half of the experiments. Even for the worst performing regional summary (PLNPCA-ward, southwest), 10% of experiments led to the 8071-dimensional emulator having lower RMSE than a one-dimensional emulator trained to this output alone.

**Figure 5.**
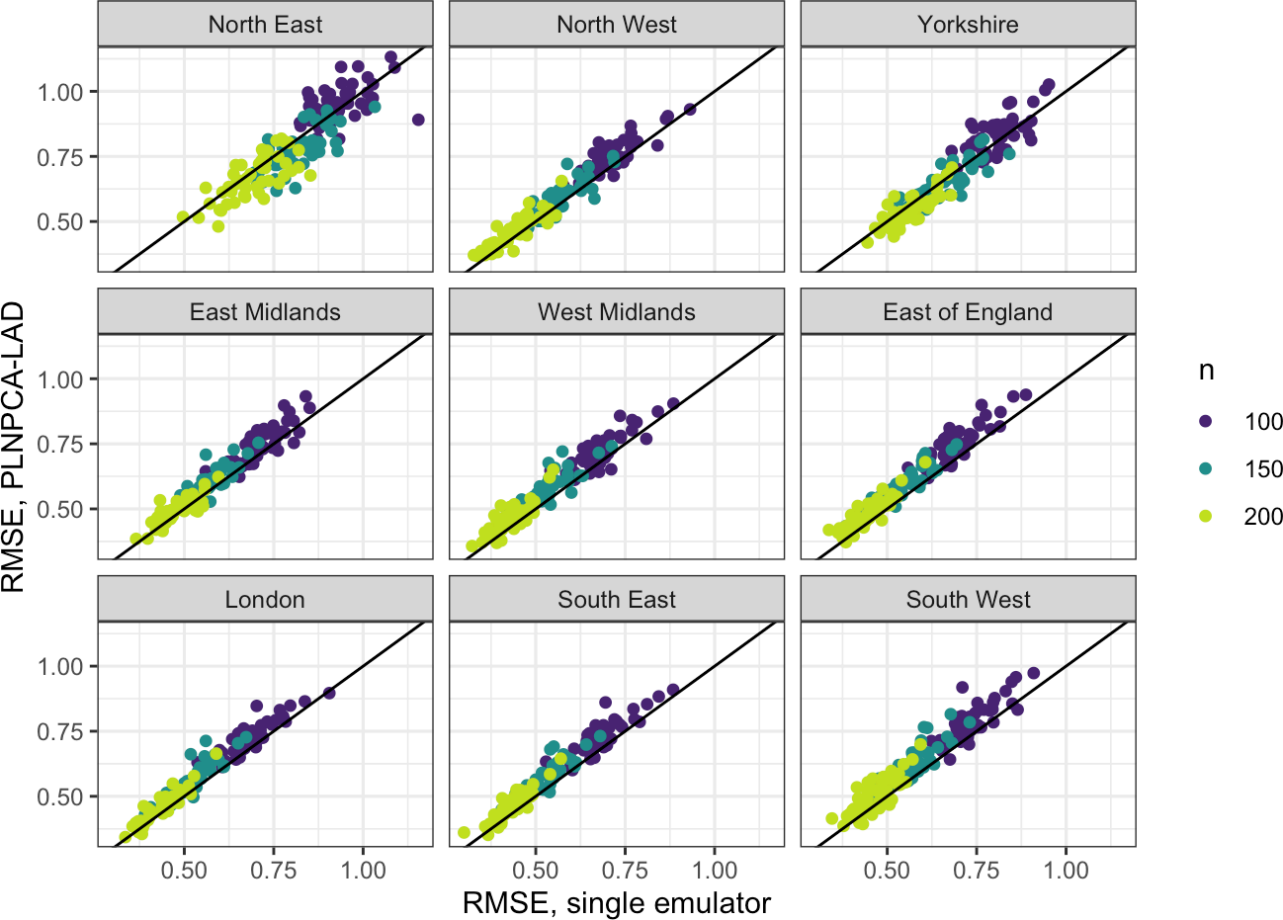
RMSE between simulator deaths and emulator predictions across the validation set for different sizes of training set (n=100,150,200), for samples from the PLNPCA-LAD emulator aggregated to a regional level compared with individually emulating each region. Each point represents a particular split into training and validation sets (50 per n).

Figure 5 gives an alternative visualization of the results from table 1, plotting the RMSE by region for all 150 splits for PLNPCA-LAD and individual hetGP emulators for the nine regions. For some regions, PLNPCA-LAD results in a small increase in RMSE; however, for others there is more variability in which emulator is more accurate. The NE region is least correlated with the overall total, generally has lower output than the other regions and is the region along with YO where there appears to be the most value gained from the PLNPCA approach. For all regions, there is at least one training set where the individual emulator is performing worse. Electronic supplementary material, fig. S40, contains a similar plot comparing the regional performance of the two PLNPCA emulators, and the results are reasonably consistent, despite needing to predict and aggregate from 8071 outputs.

## 6. Discussion

In this article, we have emulated a stochastic simulator with high-dimensional count output using a PLNPCA basis decomposition. By exploiting the PLN structure, samples from the PLNPCA emulator are high-dimensional fields of counts that encode correlations from the simulator output via a latent basis, so that emulator samples resemble the true high-dimensional simulator output. GPs are trained for the leading latent coefficients to capture signal dependent on the input parameters, with patterns due to the stochastic nature of the simulator included in the emulator variance, with samples accounting for this source of variability. Given a PLNPCA emulator, samples can be viewed across any, all or combinations of individual locations, allowing great flexibility in exploring the emulator output and effects of inputs. The example here focused on spatial output, but the method translates to other high-dimensional count outputs.

The comparison between the PLNPCA emulators trained on LAD-level (339-dimensional) and ward-level (8071-dimensional) data to emulators trained on summaries of the simulator output showed that there is not a consistently superior approach, and the results are dependent on the available training data and output of interest. Even for the overall total, inferring this from a PLNPCA emulator did not lose significant predictability on average, and was not always more accurate with a single emulator. In the comparisons here, there was usually little difference between PLNPCA-LAD and PLNPCA-ward, suggesting we can gain extra granularity without losing much predictive ability, although this may be due to the particular correlated nature of this simulator. The basis emulation scales well to high ℓ, allowing localized information to be explored while only needing a small number of GPs for the leading latent coefficients.

Achieving similar accuracy across different summaries, and in fact being better for some aspects of the output, without having to fit new emulators for each new output of interest, suggests that the PLNPCA approach should be considered for these types of simulators. Gaining the ability to more accurately predict the high-dimensional variability in the simulator, the extra time required to calculate the basis is worthwhile because we can assess a wide range of different outputs at once. Characterizing the spatial, or other high-dimensional, variability and dependencies in simulator output can be important, and in a calibration exercise, may help to avoid trading off competing biases that lead to a good fit to the total, but implausible high-dimensional output. If we have a chain of simulators and need spatial information to feed into other models and decision-making, then having an emulator that produces output with the same properties (counts, correlated, at ward level) as the true model can be important.

As with other data-driven low-dimensional representations, by definition we should be able to represent the variability in the original simulator output reasonably well, but may struggle with extrapolation, particularly when the input space is large, the number of simulations is small and the output is highly variable. If interested in calibration, we may need to more carefully design the basis so that we are able to extrapolate properly and better explore model discrepancies: are we unable to produce observations because of inadequacies of the simulator or because the basis cannot extrapolate well? We have not considered calibration here, but this is the focus of other work.

## Data Availability

Data and code are available at [35]. Supplementary material is available online [36].
